# An Ecosystem-Based Approach to Assess the Status of a Mediterranean Ecosystem, the *Posidonia oceanica* Seagrass Meadow

**DOI:** 10.1371/journal.pone.0098994

**Published:** 2014-06-16

**Authors:** Sébastien Personnic, Charles F. Boudouresque, Patrick Astruch, Enric Ballesteros, Sylvain Blouet, Denise Bellan-Santini, Patrick Bonhomme, Delphine Thibault-Botha, Eric Feunteun, Mireille Harmelin-Vivien, Gérard Pergent, Christine Pergent-Martini, Jérémy Pastor, Jean-Christophe Poggiale, Florent Renaud, Thierry Thibaut, Sandrine Ruitton

**Affiliations:** 1 Aix-Marseille University, Mediterranean Institute of Oceanography (MIO), Université de Toulon, CNRS/INSU, IRD, UM 110, Marseille, France; 2 GIS Posidonie, Pytheas Institute, Aix-Marseille University, Marseille, France; 3 Centre d'Estudis Avançats de Blanes - CSIC, Blanes, Spain; 4 Aire marine protégée de la côte Agathoise, site natura 2000, Agde, France; 5 Aix-Marseille University, Institut Méditerranéen de Biodiversité et d'Ecologie (IMBE), UMR 7263, Station Marine d'Endoume, Marseille, France; 6 Museum National d'Histoire Naturelle, UMR 7208, Station Marine de Dinard, France; 7 Equipe Ecosystèmes Littoraux, FRES 3041, University of Corsica, Corte, France; 8 Université de Perpignan, Via Domitia, Centre de Formation et de Recherche sur les Environnements Méditerranéens, UMR 5110, Perpignan, France; CSIR- National institute of oceanography, India

## Abstract

Biotic indices, which reflect the quality of the environment, are widely used in the marine realm. Sometimes, key species or ecosystem engineers are selected for this purpose. This is the case of the Mediterranean seagrass *Posidonia oceanica*, widely used as a biological quality element in the context of the European Union Water Framework Directive (WFD). The good quality of a water body and the apparent health of a species, whether or not an ecosystem engineer such as *P. oceanica*, is not always indicative of the good structure and functioning of the whole ecosystem. A key point of the recent Marine Strategy Framework Directive (MSFD) is the ecosystem-based approach. Here, on the basis of a simplified conceptual model of the *P. oceanica* ecosystem, we have proposed an ecosystem-based index of the quality of its functioning, compliant with the MSFD requirements. This index (EBQI) is based upon a set of representative functional compartments, the weighting of these compartments and the assessment of the quality of each compartment by comparison of a supposed baseline. The index well discriminated 17 sites in the north-western Mediterranean (French Riviera, Provence, Corsica, Catalonia and Balearic Islands) covering a wide range of human pressure levels. The strong points of the EBQI are that it is easy to implement, non-destructive, relatively robust, according to the selection of the compartments and to their weighting, and associated with confidence indices that indicate possible weakness and biases and therefore the need for further field data acquisition.

## Introduction

Human activities can deeply alter the environment, species composition and functioning of ecosystems. These alterations can be tracked by the use of biotic indices, i.e. species or groups of species whose function, population, or status reflect the environmental quality. Thus, biotic indices are monitored for changes in presence and abundance. The occurrence of an organism in a specific environment indicates that overall, its biological requirements are satisfied, whereas its disappearance suggests a change in the environment. Species are also monitored for changes in biochemistry, physiology or behaviour induced by environmental conditions. Biotic indices are used in terrestrial, freshwater and marine habitats, because they enable the quality of an environment to be characterized in an integrated way [1]–[7].

Biotic indices are widely used in the marine realm to **(i)** assess the quality of a water body, **(ii)** assess processes such as currents, sedimentation and climate under natural and anthropogenic forcing, and **(iii)** monitor the status of species or communities of interest, either emblematic species, indicators of some ecosystemic processes or indicators of pollution. Sometimes, key species and ecosystem engineers [8]–[10] are selected for this purpose. This is the case of the seagrass *Posidonia oceanica* [11]–[17].

In the European Union (EU), the so called Habitats Directive of 1992 (92/43/ECC) listed habitats and species that are used to designate areas (‘Natura 2000 sites’), where they are strictly protected. While the Habitats Directive also considered the marine realm, more recently, the EU Marine Strategy Framework Directive (MSFD: 2008/56/EC) established a framework for conservation in the field of marine environmental policy. The MSFD is considered to be the environmental pillar of the Integrated Maritime Policy adopted in 2010 by the European Commission (2010/477/EU). This directive established eleven criteria, based on the descriptors set out in the MSFD, to determine ‘good environmental status’: **(i)** Biological diversity is maintained, **(ii)** Introduced species are at levels that do not adversely alter the ecosystems, **(iii)** Populations of all exploited fish and shellfish are within safely biological limits, **(iv)** All elements of the food webs occur at levels capable of ensuring the long-term abundance of the species, **(v)** Human-induced eutrophication is at a minimum, **(vi)** Sea-floor integrity is at a level that ensures that the structure and functions of the ecosystems are safeguarded, **(vii)** Permanent alteration of hydrographical conditions does not adversely affect marine ecosystems, **(viii)** Concentration of contaminants are at levels not giving rise to pollution effects, **(ix)** Contaminants in fish and other seafood do not exceed levels established by Community legislation, **(x)** Properties and quantities of marine litter do not cause harm to the coastal and marine environment, and **(xi)** Introduction of energy is at levels that do not adversely affect the marine environment. The EU MSFD established a framework within which Member States agreed to take the appropriate measures to achieve or maintain good environmental status in the marine realm by the year 2020 at the latest.

A key point of the MSFD is the ecosystem-based approach. Most previous strategies only dealing with Biological Quality Elements (BQE: species and/or communities) used in the European Union Water Framework Directive (WFD) were not indicative of ecosystem status, but merely indicative of the environmental status of water bodies [18]–[22], [17], [23] for a review of the approaches for classifying and assessing quality of benthic habitats. As an example of the possible shortcomings that may arise from the WFD descriptors, a seagrass meadow characterized by normal leaf growth, shoot density and the absence of mechanical injuries would be ranked as ‘good’, even if deprived of some basic compartments of the ecosystem.

According to the MSFD, ‘good environmental status’ means that the marine environment is at a level that allows uses and activities by current and future generations, i.e. the structure, functions and processes of the constituent marine ecosystems, together with the associated physiographic, geographic, geological and climatic factors, allow those ecosystems to function fully and to maintain their resilience to human induced environmental change. Overall, marine species and marine habitats are protected, human-induced decline of biodiversity is prevented and diverse biological components function in balance.

While the ecosystem-based approach constitutes the background and the guidelines of the MSFD, the pristine state of an ecosystem is not clearly defined. Obviously, such a notion is very complex and prone to anthropocentrism; reference conditions, as observed in areas distant from known human impact, may constitute a more realistic notion. Here we try to define: **(i)** how the status of an ecosystem can be measured and how much its current status differs from ‘reference conditions’, and **(ii)** which parameters we need to monitor to ascertain these targets. The *Posidonia oceanica* seagrass meadow was chosen for this attempt because: **(i)** it is widely present in almost the whole of the European Mediterranean; **(ii)** it is the only marine ecosystem considered as ‘priority habitat’ by the EU Habitats Directive; **(iii)** its functioning is relatively well known [11], [24]–[30]; and **(iv)** like many seagrass ecosystems in the world ocean, *P. oceanica* meadows have been impacted or lost under the influence of direct and indirect effects of human activities and are therefore regarded as threatened [31]–[33]. Similar ecosystem-based approaches have been attempted for fisheries (e.g. [34]–[38], [40], [41]) and for the management and conservation of ecosystem services (e.g. [34], [41], [42]).

## Materials and Methods

### The conceptual model

**Figure 1:**
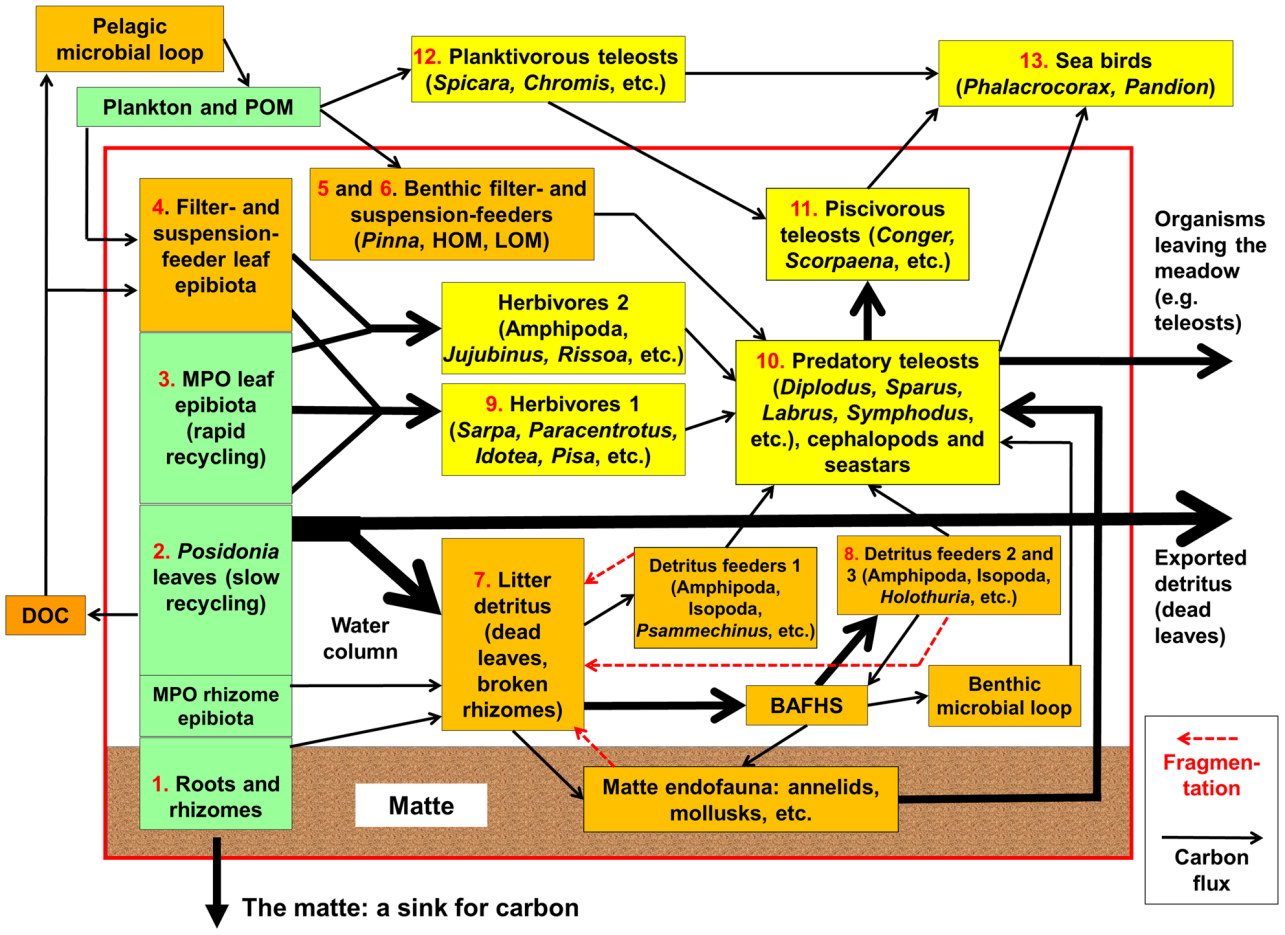
Conceptual model of the functioning of *Posidonia oceanica* seagrass ecosystem. For functional compartments and box numbers, see text. Primary producers are in green; filter-feeders, suspension-feeders, litter, detritus feeders, Dissolved Organic Carbon (DOC) and microbial loops are in orange; predators (including herbivores) are in yellow. The width of the arrows roughly represents the importance of the carbon flow. The proper *P. oceanica* ecosystem is included within the red rectangle. MPO: Multicellular Photosynthetic Organisms. POM: Particulate Organic Matter. From C.F. Boudouresque, unpublished.

*Posidonia oceanica* (L.) Delile is a seagrass species (Magnoliophyta, kingdom Archaeplastida) endemic to the Mediterranean Sea [43], which dwells in the sublittoral zone, from mean sea level down to 30 to 40 m depth, depending upon water transparency [27], [28], [32]. Due to the length, up to 120 cm, and density of the leaves, the seagrass canopy decreases water movement and traps sediments [44]–[47]. Rhizomes resist burial by vertical growth, so that the sea bottom slowly rises. Within the sediment, the deeper parts of the rhizomes, attached leaf sheaths and roots die, but their decay is extremely slow, so that they can persist for millennia [24], [27]. The terrace constituted by live and dead intertwined rhizomes, together with the sediment, which fills the interstices, is named ‘matte’ [27], [48], [49]. When *P. oceanica* dies, the matte persists (hereafter ‘dead matte’), since the decay of the rhizomes proceeds extremely slowly [44], [50]. *P. oceanica* is the engineer of an ecosystem of major importance in the Mediterranean Sea [27], [28]. A conceptual model of the functioning of the *P. oceanica* ecosystem has been proposed [11], [27], [28]. Here, we use an updated version of this conceptual model (Fig. 1; C.F. Boudouresque, unpublished).

The simplified conceptual model of the functioning of the *P. oceanica* ecosystem in the north-western Mediterranean Sea used in the present study (Fig. 1) encompasses the following compartments (boxes); these compartments are listed hereafter and detailed later on:

*Posidonia oceanica* roots and rhizomes (box 1).
Multicellular Photosynthetic Organisms (MPOs) epibiotic on *P. oceanica* rhizomes.
*P. oceanica* leaves (box 2).
MPO leaf epibiota (box 3).
Dissolved Organic Carbon (DOC).
Pelagic microbial loop.
Filter- and suspension-feeder leaf epibiota (box 4).
Filter- and suspension-feeder benthic epibiota on *P. oceanica* rhizomes: the bivalve *Pinna nobilis* (box 5) and other species, e.g. bryozoans and ascidians (box 6).
The litter detritus (essentially dead *P. oceanica* leaves and some broken rhizomes) (box 7).
Detritus feeders 1, e.g. Amphipoda, Isopoda (crustaceans) and *Psammechinus microtuberculatus* (sea urchin).
Detritus feeders 2 and 3, e.g. Amphipoda, Isopoda (crustaceans) and *Holothuria* spp. (sea cucumber) (box 8).
Decomposers, namely *Bacteria*, *Archaea*, Fungi and heterotrophic stramenopiles (BAFHS) such as Labyrinthulomycota and Oomycota.
The benthic microbial loop.
The matte endofauna, e.g. annelids and mollusks.
Herbivores 1, e.g. *Sarpa salpa* (teleost), *Paracentrotus lividus* (sea urchin), *Idotea* spp. and *Pisa* spp. (crustaceans) (box 9).
Herbivores 2, e.g. Amphipoda (crustaceans), *Jujubinus* spp. and *Rissoa* spp. (gastropods).
Predatory teleosts (e.g. *Diplodus* spp., *Sparus aurata*, *Labrus* spp. and *Symphodus* spp.), cephalopods and seastars (e.g. *Marthasterias glacialis*) (box 10).
Piscivorous teleosts, e.g. *Conger conger*, *Scorpaena* spp. and *Serranus* spp. (box 11).
Planktivorous teleosts of the water column, e.g. *Spicara* spp. and *Chromis chromis* (box 12).
Sea birds, e.g. *Phalacrocorax aristotelis* ssp. *desmarestii* and *Pandion haliaetus* (box 13).
Plankton (photosynthetic plankton and zooplankton) and non-living Particulate Organic Matter (POM).

### Considered functional compartments (boxes)

Whenever possible, non-destructive methods were chosen to measure the parameters of the status of the functional compartments, as suggested by [51]. In the absence of further indications, measures are performed at a depth of ca. 15 meters (10 to 20 m), which is then considered as representative of the whole depth range [14], [52]. The season of the sampling is indicated only for compartments which present seasonal variability. In the ranking of the status of the selected compartments (boxes), two cases were encountered; **(i)** a steady trend of the parameter, from very good to a low quality state; **(ii)** an upward/downward slope parameter, when the very good state corresponds to intermediate values.

#### *Posidonia* roots and rhizomes

(box 1, Fig. 1). This compartment was estimated by the growth rate of vertical (orthotropic) rhizomes. The matte compartment results in carbon sequestration within the matte, which acts as a carbon sink [27], [44], [53], [54] and was measured by means of lepidochronology [55], [56]. Lepidochronology describes the annual cycle of leaf formation. A cycle includes a suite of dead leaf bases (called dead sheaths or scales) of increasing then decreasing thickness. An average of 7.5 leaves (generally between 6 and 9) are produced every year [57]–[60]. We considered that high and low rhizome growth rates are indicative of over sediment input or of deficit in sediment, respectively (Table 1). The highest growth rate of an orthotropic rhizome is 7 cm year^−1^; higher sedimentation rates result in the death of the buried leaf bundle [44], [61]. In contrast, deficit in sediment results in bare, non-sediment protected rhizomes, which are hence very vulnerable to storms, anchoring and trawling [27]. The nature of the substratum, namely meadows settled on rock, sand or matte, only slightly influences the growth rate of rhizomes [62] and no major differences were found at the community level [63] so that it was not necessary to adapt the scale. Thirty random *in situ* measures (growth of the rhizome corresponding to the last 8 dead leaf bases) are recommended. The obtained value was multiplied by 1.5 in order to take into account the fact that the rhizome continues to grow slowly during the following two years (Gérard Pergent, unpublished data).

**Table 1:** Relative weighting of each functional compartment (boxes; see Fig. 1 for box number) and ranges of each parameter defined for each grade of ecosystem status.

Box number	Functional compartment	Weighting (W)	Parameter	4	3	2	1	0
1	Roots and rhizomes (‘rhizomes’)	3	Growth of orthotropic rhizomes (mm a^−1^)	9 to 19	20 to 40	3 to 8	>40	<3
2	*Posidonia oceanica* leaves (‘leaves’)	5	- Density (shoots m^−2^)	≥490	489 to 370	369 to 250	249 to 130	<130
			- Cover (%)	>80	80 to 61	60 to 41	40 to 20	<20
3–4	MPOs, filter- and suspension-feeders leaf epibiota (‘leaf epibiota’)	4	Biomass (g DM shoot^−1^) (only the 2 oldest - external - leaves)	0.3 to 0.7	0.1 to 0.2	0.8 to 1.5	<0.1	>1.5
5	Benthic filter-feeder: *Pinna nobilis* (bivalve) (‘Pinna’)	2	Density (individuals 100 m^−2^)	>3.0	3.0 to 1.1	1.0 to 0.6	0.5 to 0.1	<0.1
6	Other benthic filter- and suspension-feeders (‘HOM LOM’)	2						
	- HOM		- Density (m^−2^)	<0.1	0.1 to 0.9	1.0 to 1.9	2.0 to 5.0	>5.0
	- LOM		- Density (m^−2^)	>10.0	10.0 to 5.1	5.0 to 1.1	1.0 to 0.1	<0.1
7	Litter detritus: dead leaves and broken rhizomes (‘litter’)	2	g DM m^−2^	>350	350-251	250-151	150-51	≤50
8	Detritus-feeders 2 and 3 (*Holothuria* spp.) (‘Holothuria’)	2	Individuals 10 m^−2^	1.0 to 4.9	0.2 to 0.9	5.0 to 24.9	<0.2	≥25.0
9	Herbivores 1 (‘herbivores’)	5	- Density of *Paracentrotus lividus* (individuals m^−2^)	1.0 to 4.9	0.1 to 0.9	5.0 to 9.9	<0.1	≥10
			- Grazing index (% leaves)	30 to 59%	5 to 29%	60 to 95%	<5%	>95%
10	Predatory teleosts, cephalopods and seastars (‘predators’)	5	kg teleosts WM 100 m^−2^	>1.5	1.5 to 1.1	1.0 to 0.6	0.5 to 0.3	<0.3
11	Piscivorous teleosts (‘piscivores’)	5	kg teleosts WM 100 m^−2^	>1.0	1.0 to 0.6	0.5 to 0.3	0.2 to 0.1	<0.1
12	Planktivorous teleosts (‘planktivores’)	3						
	- Zooplankton feeders		- kg teleosts WM 100 m^−2^	>3.0	3.0 to 1.6	1.5 to 0.8	0.7 to 0.3	<0.3
	- Omnivores		- kg teleosts WM 100 m^−2^	>3.0	3.0 to 1.6	1.5 to 0.8	0.7 to 0.3	<0.3
9–12	All teleosts (‘SRDI’)	3	Specific Relative Diversity Index (SRDI)	>10	10 to 8	7 to 5	4 to 3	<3
13	Sea birds	1						
	- *Phalacrocorax* spp.		- Distance to the nearest nesting site (km)	<4	4 to 7	8 to 12	13 to 17	>17
	- *Pandion haliaetus*		- Distance to the nearest nesting site (km)	<4	4 to 7	8 to 12	13 to 17	>17

Between inverted commas: abbreviated name of the functional compartment in tables 4 and 7. DM: dry mass, including calcium carbonate. HOM: indicators of high level of organic matter. LOM: indicators of low level of organic matter. MPO: Multicellular Photosynthetic Organisms. POM: Particulate Organic Matter. WM: wet mass. Status: 4 (very good) through 0 (very low).

#### *Posidonia* leaves

(box 2, Fig. 1). A shoot is a rhizome tip with a bundle of living leaves. Shoot density is correlated with annual leaf primary production at local scale (patch) [64], [65]. Primary production is a basic parameter for the functioning of the *P. oceanica* ecosystem. It was estimated by the number of shoots per square meter, measured within a small square frame (0.16 m^2^ [66], [67]) with at least 20 random replicates [5]. At a less local scale, the cover rate of the meadow is rarely 100%: it is broken by more or less extensive patches of either sand or dead matte, which reduce the overall cover. Cover rate was estimated by visual observation *via* a see-through plastic slide [68], [69] or *via* vertical photographs [67], [70]. Thirty random measures are recommended. Low cover rate is believed to characterize a poor condition of the meadow [5]. Some types of *P. oceanica* meadow, e.g. the hill meadow and the striped meadow, exhibit a low cover rate while in pristine state; these types of meadows do not, however, occur in the study area [27]. The final *P. oceanica* leaf index, for a given site, was the arithmetical mean between the density index, and the cover index.

#### MPOs, filter- and suspension-feeder leaf epibiota

(boxes 3 and 4, Fig. 1). *P. oceanica* leaf epibiota, both primary producers (MPOs and diatoms) and animals, share the same habitat (the leaf surface) and are usually co-consumed by the same species. In addition, some consumers eat simultaneously epibiota and the supporting leaf [68], [71]. For these reasons, epibiota will be considered here as a single compartment. The colonisation of leaves by epibiota is a function of leaf age, the youngest leaves, in the center of the shoot, being less colonized, while the oldest, external leaves are the most colonized; in addition, leaf tips are more colonized than the lower parts of the leaves [72]. Leaf epibiota cover is believed to provide information on water quality, especially nutrient concentration in seawater [5], [73], [74]; however, it also reflects the herbivore pressure, epibiota biomass decreasing when macrograzer abundance increases [26]. By convention, the epibiota biomass was estimated on the two oldest (external) leaves, in July, on 30 randomly localized shoots (Table 1).

#### Filter- and suspension-feeder epibiota on *P. oceanica* rhizomes

(boxes 5 and 6, Fig. 1). A number of benthic filter- and suspension-feeders dwell on *P. oceanica* rhizomes, sometimes within the matte. They belong to bryozoans, hydroids, sponges, annelids (e.g. *Sabella spallanzani*), ascidians (e.g. *Halocynthia papillosa*, *Phallusia mammillata*, *P. fumigata*), gastropods and bivalves (such as the fan mussel *Pinna nobilis*) [75]–[79]. *Pinna nobilis* (box 5) density was estimated along 20 transects 10-m long and 1-m wide. Filter- and suspension-feeders other than *P. nobilis* (box 6) are indicators either of: **(i)** high level of organic matter in the water (hereafter HOM; e.g. *Sabella spallanzani*, *Phallusia mammillata*, *P. fumigata*, Didemnidae [80]); or **(ii)** low level of organic matter (hereafter LOM; e.g. bryozoans, sponges, *Halocynthia papillosa*, *Antedon mediterranea* [80]). HOM and LOM indicators are assessed within the same quadrats as the sea urchin *P. lividus* (see below, box 9). For colonial species, the number of colonies was taken into account, as suggested by [81]. For non-colonial species, the number of individuals was counted. Only individuals and colonies over 5 cm in diameter and/or height were considered. The final filter- and suspension-feeder epibiota (other than *P. nobilis*; box 6) on rhizomes index, for a given site, was estimated as the arithmetical mean between HOM and LOM indices.

#### Litter detritus

(box 7, Fig. 1). The litter detritus mass corresponds essentially to shed dead *P. oceanica* leaf blades and the epibiota they harbour, and some broken rhizomes. It therefore represents a kind of necromass [82]. Drift MPOs, exported from sublittoral reef habitats, can also occur within the litter. The litter detritus mass was estimated in July, within 5 randomly localized 0.1 m^2^ quadrats; the litter was sucked up by an underwater vacuum cleaner. Litter detritus were dried at 50°C in an oven to constant weight.

#### Detritus-feeders 2 and 3

(box 8, Fig. 1). Detritus-feeders constitute a complex set of compartments. Here, the macro-detritus feeders *Holothuria* spp. were used as a proxy of detritus-feeders 2 and 3, as they are easy to count. Several species can be present, e.g. *H. polii* and *H. tubulosa* [83]. The abundance of *Holothuria* spp. was measured within the same quadrats as the sea urchin *P. lividus* (see below, box 9).

#### Herbivores 1

(box 9, Fig. 1). Macro-herbivores considered in this compartment were the sea urchin *Paracentrotus lividus* and the teleost *Sarpa salpa*, at 5 meters depth or, if the meadow is not present at this depth, at the upper limit of the meadow. They consume *P. oceanica* leaves, together with their epibiota, if present [68], [71], [84]. Other herbivores graze *P. oceanica* leaves, such as the spider crabs *Pisa* spp. and the isopod *Idotea hectica* [27], [84], but their reduced size and habit make them more difficult to quantify and they were not considered. The abundance of *P. lividus* was assessed within 1-m^2^ quadrats, with 30 replicates randomly localized. The census only considered individuals >3 cm (test diameter without spines), because small individuals can be hidden within the matte, generating bias in the census. A second proxy of the macro-herbivore pressure was the grazing index, i.e. the percentage of intermediate and adult leaves (*sensu* [85], [86]) exhibiting bite scars due to *S. salpa* (all the intermediate and adult leaves of 30 shoots randomly localized). Bite scars were carefully distinguished from broken leaves, the latter being related to hydrodynamism. Most bite scars are due to the *S. salpa* browsing, and they are easy to distinguish from those due to other macro-herbivores [26], [49]. The final macro-herbivore index was, for a given site, the arithmetical mean between the *Paracentrotus* index and the grazing index.

#### Predatory teleosts and cephalopods, piscivorous teleosts, planktivorous teleosts

(boxes 9 in part, 10, 11 and 12, Fig. 1). Teleost fishes associated with seagrass beds occupy different positions in and above the canopy during the day and at night, spend more or less time in this habitat depending on their life cycle, and naturally fluctuate in abundance and biomass with depth, season and years [87]–[90]. They participate actively in the functioning of the *P. oceanica* ecosystem (Fig. 1), but the perception of the composition and trophic structure of their assemblages largely depends on the methodology used [91]. Moreover, species richness, abundance and biomass of teleosts are favoured by the ecotones induced by the presence of rocky or sandy substrates in the middle of *Posidonia* beds. So these variations and methodological biases have to be taken into account to when measuring these compartments in order to assess the ‘environmental status’ of a site. These compartments were estimated *via* visual censuses at a standardized day time (10:00 to 16:00 UT) during the warm season (summer-autumn), preferentially in uniform beds (at least a long way from rocky substrates). All teleosts were counted within ten linear and 5-m wide transects, each census lasting 5 minutes. Around 50 m were covered so that each transect represents a surface area of nearly 250 m^2^. Total length (at the nearest 2 cm) of individuals and the number of individual per species were noted. The Specific Relative Diversity Index (SRDI) is the mean number of species met with per transect. These data enable calculation of **(i)** predator biomass (predatory teleosts other than piscivorous and planktivorous; box 10), **(ii)** top predator biomass (piscivorous teleosts; box 11), **(iii)** planktivorous teleost biomass (box 12) and **(iv)** the Specific Relative Diversity Index (SRDI). The planktivorous teleost biomass is divided into 2 categories: the exclusive zooplankton feeders (*Chromis chromis*, *Spicara smaris*, *S. maena*, *Atherina* spp.) and the omnivorous feeders, which consume both zooplankton and POM (*Boops boops*, *Oblada melanura*). Some top predators (e.g. *Conger conger*, *Scorpaena* spp.) are active only during night time while *Serranus* spp. are active by day and thus more present in our visual counts. Some predators (e.g. *Symphodus rostratus*), though diurnal, are often hidden within the *P. oceanica* leaf canopy. As a result, their counts were underestimated to a greater or lesser extent. The parameter ranges within the status scale (Table 1) took into account these biases.

#### Sea birds

(box 13, Fig. 1). Most sea birds do not directly interact with the *P. oceanica* ecosystem, as they feed on offshore pelagic species, such as *Larus* spp. and *Puffinus* spp. The only exceptions are shags *Phalacrocorax* spp. and the osprey *Pandion haliaetus*. Shags can dive down to the benthic seagrass meadow; they mainly feed on pelagic planktivorous teleosts (e.g. *Spicara smaris*, *Chromis chromis*), but benthic teleosts (e.g. *Diplodus* spp., *Lithognathus mormyrus*, *Scorpaena notata*, *Serranus scriba* and *Symphodus mediterraneus*) account for 35% of the diet [92]–[94]. Osprey is an opportunistic fish-eating bird of prey. In Corsica, which harbours the only population within the study area, it mainly consumes mugilids (73% of the captures), together with *Diplodus* spp. (13%) and *Sarpa salpa* (11%) [95]–[97]. The sea bird compartment was estimated *via* the distance of the nearest *Phalacrocorax* spp. and *Pandion haliaetus*, respectively, nesting sites from the study site. As far as shags are concerned, the mean maximum foraging range is 16 km [95]–[97] (Table 1).

Some of the above-mentioned compartments correspond to inputs into the *P. oceanica* ecosystem from the pelagic habitat: plankton, POM and planktivorous teleosts. Outputs are also to be considered: **(i)** ca. 15–30% of the net primary production (NPP) corresponds to roots, rhizomes and dead sheaths buried and sequestrated within the matte [24], [28], [98], [99]; **(ii)** ca. 6 to 50% of the NPP represents dead leaves that are exported as detritus towards beaches and adjacent habitats [28], [31], [98], [100], [101]; and **(iii)** a number of organisms (e.g. teleosts, crustaceans) leave the meadow, either temporarily, to feed in adjacent habitats, or permanently, to reach their adult quarters [87], [102]–[104].

### The Ecosystem-Based Quality Index (EBQI)

The rationale governing our ecosystem-based approach is trying to quantify and assess each compartment (box) of the conceptual model by means of a set of parameters, to balance their relative weighting and by using a simple algorithm to calculate a rank for the ecosystem status within a given area, matching the five Ecological Statuses of the Water Framework Directive (WFD), from bad to high. A great variety of parameters are available for the assessment of each compartment (box). Many of them are redundant. Others have been poorly used so that data are not available for most areas. For this reason a limited set of relevant parameters was selected for a restricted set of compartments.

Each parameter was assessed by means of a semi-quantitative scale (4 through 0), from very good (4) to very low (0). Calibration of the scale was based upon the available literature (e.g. [105]), including grey literature and expert judgment (the personal knowledge of the authors) based upon a Delphi process [106]. The highest grade (4) corresponds to the ecosystem status in the best-protected areas of well implemented MPAs, e.g. the Medes Islands reserve (Catalonia, Spain), the Port-Cros National Park (continental France), the Scandola reserve and the Bouches de Bonifacio reserve (Corsica, France).

Boxes were balanced, according to their relative weighting (W) in the ecosystem functioning, from 5 (highest weighting) to 1 (lowest weighting). The general principle in the ranking of the weighting of a box was that the boxes localized at the very base (bottom up control by primary producers), the herbivores (box 9) and the boxes localized at the very top (top down control via cascade effect) of the model were regarded as of major importance (with the exception of box 13), while intermediate ones were less weighted. Wasp-waist control has not been evidenced in that ecosystem [11], [107]. The grade of each considered box was given by multiplying its status S (0 through 4) and weighting W (1 through 5), and therefore they are graded from 0 through 20. The grades of all considered boxes were added up, which gave the final grade of the ecosystem status (Ecosystem Based Quality Index, hereafter EBQI) at a given site. For practical purposes, the EBQI was converted to a scale from 0 to 10:
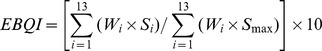
Where: W_i_ is the weighting of the box i, S_i_ the status of the box i, S_max_ the highest possible grade ( = 4) for a box and i is the number of the box (1 through 13).

Five ecological status classes, from high to bad, according to the practice of the WFD, were delineated: bad (EBQI<3.5), poor (3.5≥EBQI<4.5), moderate (4.5≥EBQI<6), good (6.0≥EBQI<7.5) and high (EBQI≥7.5).

Since the box weightings were supported by partly subjective arguments (see above), we aimed to analyse the effect of the weighting choice on the EBQI and the ranking of the sites. In order to achieve our aim, we perturbed each weighting value and determined the new ranking obtained with the perturbed weightings. The perturbation on each weighting was obtained as follows. We first defined the maximum amplitude of the perturbation for all weightings and we then defined, for each weighting, a random perturbation, according to a uniform law between 0 and the maximum amplitude of perturbations. We then added or subtracted this perturbation term to or from the corresponding weighting. If the new weighting was less than 1, it was then set as equal to 1. If the new weighting was greater than 5, it was then set as equal to 5. We ended up with weightings between 1 and 5, close to the original ones if the maximum perturbation amplitude was low, and randomly selected otherwise. This has been calculated as follows: the perturbation method described previously was repeated 1,000 times and, for each site, we determined whether the rank of the site was the same as the initial one or if it had changed. An index of similarity was produced for each site, which was equal to 100% when the rank of the site was always unchanged after perturbation and 0% if it always changed.

For each compartment (box) status at each site, a Confidence Index (CI) was proposed (Table 2). The reason for the CI is **(i)** that data for one or several compartments may be missing or of poor quality in some sites, **(ii)** the reliability of available data may be different between compartments and sites, and **(iii)** it is worth drawing the attention of managers and scientists to those compartments that are poorly known and which merit further field studies. The grade of each considered compartment was given by its CI (0 through 4) and by its weighting (1 through 5), and therefore they are graded from 0 through 20. The grades of all considered compartments were added up, which gave the final mark of the CI at a given site. For practical purposes, the CI was converted to a scale from 0 to 4:
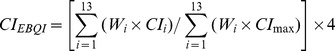
Where W_i_ is the weighting of the box i, CI_i_ the Confidence Index of the box i, CI_max_ the highest possible Confidence Index ( = 4) for a compartment (box) and i is the number of the box (1 through 13).

**Table 2:** Criteria to assess the Confidence Index (CI) of the status of a compartment (box).

CI	Criteria
4	Field data available, recent and suitable with the recommended methods
3	Field data recent, partially completed with expert judgment
2	No quantitative field data but recent expert judgment
1	No quantitative field data, but ancient expert judgment
0	No quantitative field data and no suitable expert judgment

In order to test the efficiency of the proposed method, it was applied to seventeen sites (Table 3) with a variety of available data (published, unpublished, expert judgment). A site is defined as a *P. oceanica* meadow, from its upper limit down to the lower limit, covering in the order of a dozen to several hundred hectares. The sites are localized in the north-western Mediterranean Sea, including continental France (French Riviera, Provence, French Catalonia), Corsica, Balearic Islands and Spanish Catalonia. This area is considered as homogenous and is consistent with the marine subregions, as defined in the MSFD. These sites also cover of a wide range of human pressures, from lesser impact, within well implemented Marine Protected Areas (MPAs), such as the Port-Cros National Park, to highly impacted areas due to different disturbance and/or stress sources (pollution, overfishing, fish farms, port facilities, anchoring and mooring; see table 3).

**Table 3:** Sites used to test the proposed ecosystem-based approach to assess the status of the *P. oceanica* ecosystem. MPA: Marine Protected Area. NTZ: No Take Zone.

Site	Region	Protection status	Pressure
Espardell	Balearic Islands (Spain)	MPA, Natura 2000	Artisanal fishery
Sitges	Spanish Catalonia	Natura 2000	Pollution, artisanal and recreational fishery, sedimentation
Tossa de Mar	Spanish Catalonia	Natura 2000	Artisanal and recreational fishery
Medes Islands	Spanish Catalonia	MPA, NTZ, Natura 2000	River mouth
Peyrefite Bay	French Catalonia	MPA^a^	Artisanal fishery, anchoring^b^
Niolon (Côte Bleue)	Provence (France)	MPA, Natura 2000	River mouth, artisanal fishery, trawling
Prado Bay, Marseilles	Provence (France)		Coastal development, artisanal fishery, nutrients
Plateau des chèvres, Marseilles	Provence (France)	MPA^c^, Natura 2000	Artisanal fishery, sewage outfall,
Saint Cyr Bay	Provence (France)		Coastal development, artisanal fishery, sewage outfall
Gulf of Giens	Provence (France)		Sewage outfall, artisanal fishery, trawling
Porquerolles Island, northern coast	Provence (France)	MPA, Natura 2000	Trawling, artisanal fishery, anchoring
Porquerolles Island, southern coast	Provence (France)	MPA, Natura 2000	Trawling, artisanal fishery
Bagaud Pass, Port-Cros Island	Provence (France)	National Park, MPA, Natura 2000	Artisanal fishery, anchoring
Port-Cros Island, southern coast	Provence (France)	National Park, MPA, Natura 2000	Artisanal fishery
Villefranche-sur-Mer Bay	French Riviera		Coastal development, sewage outfall artisanal fishery, anchoring
Scandola, Elbu Bay	West Corsica (France)	MPA, Natura 2000	Artisanal fishery
Valincu Gulf	West Corsica (France)		Artisanal fishery

a This area is close to the Natural Marine Reserve of Cerbère-Banyuls. Since October 2011, this area has been included within a natural marine park (‘Parc naturel marin du golfe du Lion’).

b Since 2010, anchoring is banned.

c Since May, 2012, this area has been included within a National Park (‘Parc national des Calanques’). However, it is unlikely that this new status would have already resulted in a perceptible effect.

## Results

### EBQI assessment

The results of the EBQI application for assessing the status of the *P. oceanica* ecosystem functioning in the sites across the NW Mediterranean area (Table 3) are presented in Table 4. An example of calculation of the EBQI is given (Table 5). The content of Table 4 is based upon unpublished observations from the authors of the present article, together with some published data [108]–[111] and whenever necessary (CI<4) on expert judgment; when several co-authors of the present work were involved in the assessment of a site, the Delphi method [106] was used for assessing the status of each compartment (box). According to the EBQI, the 17 study sites were placed within five Ecological Status classes, from Bad to High:

**Table 4:** Ecosystem status (EBQI) and Confidence Index (CI) for each sampling site, and scale value and CI of each parameter used to obtain them.

Box	1 Rhizomes	2 Leaves	3–4 Leaf epibiota	5 Pinna	6 HOM LOM	7 Litter	8 Holothuria	9 Herbivores	10 Predators	11 Piscivores	12 Planktivores	9–12 (SRDI)	13 Sea birds	EBQI and (CI)
Espardell	4(0)	4(4)	3(3)	3(4)	3(0)	3(0)	3(0)	3(3)	1(4)	1(4)	1(4)	3(4)	2(3)	6.4(2.9)
Sitges	2(0)	0(4)	0(3)	0(3)	2(0)	2(0)	2(3)	3.5 (3.5)	0(3)	0(3)	1(3)	0(3)	0(3)	2.3(2.7)
Tossa de Mar	2(3)	3(4)	4(4)	0(3)	2(0)	2(0)	4(2)	3(2.5)	2(4)	0(4)	2(4)	3(4)	1(2)	5.6(3.2)
Medes Islands	2(3)	3.5(4)	4(4)	3(3)	2(0)	2(0)	2(0)	2.5(3)	4(2)	4(2)	3(2)	4(2)	2(2)	7.9(2.4)
Peyrefite Bay	2(0)	3.5(4)	2(0)	4(4)	2(0)	2(0)	2(0)	2(4)	3(3)	1(3)	1.5(3)	4(3)	0(2)	5.8(2.3)
Niolon	2(2)	2.5(4)	2(2)	0(2)	1.5(2)	1(0)	3(0)	2(1.5)	1(2)	0(0)	2(0)	2(2)	1(4)	3.9(1.7)
Prado Bay	2(4)	2.5(3)	2(2)	0(2)	2.5(2)	2(2)	3(2)	2.5 (1.5)	3(2)	1(2)	1.5(2)	3(2)	2(4)	5.3(2.3)
Plateau des Chèvres	2(4)	2.5(4)	4(3)	0(2)	1.5(2)	2(2)	3(2)	2.5 (1.5)	2(4)	1(4)	0.5(2)	2(4)	2(2)	5.0(3.1)
Saint-Cyr Bay	1(2)	3(3)	2(0)	1(0)	2(0)	2(2)	2(0)	2(0)	2(0)	2(0)	2(0)	2(0)	0.5(4)	4.9(0.7)
Gulf of Giens	3(4)	4(4)	2(1)	2(0)	2(0)	1(0)	3(2)	1.5(2)	1(4)	0(2)	1(2)	1(3)	0.5(4)	4.3(2.4)
Porquerolles Island northern coast	3(3)	2(4)	3(3)	2(3)	2(3)	0(2)	1(2)	1.5(1)	1(2)	1(2)	2(2)	2(2)	1(4)	4.3(2.4)
Porquerolles Island southern coast	3(3)	4(5)	4(3)	3(3)	3(3)	3(2)	2(2)	2(1)	2(2)	2(2)	3(2)	3(2)	1(4)	6.9(2.6)
Bagaud Pass, Port-Cros Island	4(2)	3(4)	2(4)	4(4)	3(2)	4(2)	4(2)	3(2.5)	3(2)	2(2)	3(2)	4(2)	1(4)	7.6(2.6)
Port-Cros Island southern coast	4(0)	4(4)	4(3)	4(2)	3(2)	4(0)	3(0)	3.5(0)	4(2)	4(0)	3(2)	4(2)	1.5(4)	9.3(1.6)
Villefranche-sur-Mer Bay	2 (1)	1.5 (4)	2(1)	1(2)	3(2)	0(0)	0(2)	2(1)	3(0)	2(1)	1.5(1)	4(0)	0(4)	4.8(1.3)
Scandola, Elbu Bay	4(4)	3(4)	1(3)	4(4)	3(2)	2(2)	2(2)	2(1.5)	2(2)	1(2)	1.5(2)	3(3)	4(4)	5.7(2.6)
Valincu Gulf	(4.4)	3(4)	2(3)	2(2)	2(0)	2(2)	3(2)	2(0)	2(0)	2(0)	2(0)	2(0)	1(0)	5.4(1.4)

Boxes 1 through 13 and SRDI. The status and the Confidence Index (in brackets; CI) is indicated for each box. EBQI ranges from 0 to 10, while the CI is graded from 0 to 4).

**Table 5:** Example of calculation of the EBQI at the site Espardell (Balearic Islands).

Box number	Functional compartment	Weighting (W)	Parameter	Status and mean status when 2 parameters (S)	CI	Status grade: W×S	CI grade: W×CI
1	Roots and rhizomes (‘rhizomes’)	3	Growth of orthotropic rhizomes (mm a^−1^)	4	0	12	0
2	*Posidonia oceanica* leaves (‘leaves’)	5	- Density (shoots m^−2^)	(4)	(4)	(20)	(20)
			- Cover (%)	(4)	(4)	(20)	(20)
				4	4	20	20
3–4	MPOs, filter- and suspension-feeders leaf epibiota (‘leaf epibiota’)	4	Biomass (g DM shoot^−1^)	3	3	12	12
5	Benthic filter-feeder: *Pinna nobilis* (bivalve) (‘Pinna’)	2	Density (individuals 100 m^−2^)	3	4	6	8
6	Other benthic filter- and suspension-feeders (‘HOM LOM’)	2					
	- HOM		- Density (m^−2^)	(3)	(0)	(6)	(0)
	- LOM		- Density (m^−2^)	(3)	(0)	(6)	(0)
				3	0	6	0
7	Litter detritus: dead leaves and broken rhizomes (‘litter’)	2	g DM m^−2^	3	0	6	0
8	Detritus-feeders 2 and 3 (*Holothuria* spp.) (‘Holothuria’)	2	Individuals 10 m^−2^	3	0	6	0
9	Herbivores 1 (‘herbivores’)	5	- Density of *Paracentrotus lividus* (individuals m^−2^)	(3)	(3)	(15)	(15)
			- Grazing index (% leaves)	(3)	(3)	(15)	(15)
				3	3	15	15
10	Predatory teleosts, cephalopods and seastars (‘predators’)	5	kg teleosts WM 100 m^−2^	1	4	5	20
11	Piscivorous teleosts (‘piscivores’)	5	kg teleosts WM 100 m^−2^	1	4	5	20
12	Planktivorous teleosts (‘planktivores’)	3					
	- Zooplankton feeders		- kg teleosts WM 100 m^−2^	(1)	(4)	(3)	(12)
	- Omnivores		- kg teleosts WM 100 m^−2^	(1)	(4)	(3)	(12)
				1	4	3	12
9–12	All teleosts (‘SRDI’)	3	Specific Relative Diversity Index (SRDI)	3	4	9	12
13	Sea birds	1					
	- *Phalacrocorax* spp.		- Distance to the nearest nesting site (km)	(4)	(3)	(4)	(3)
	- *Pandion haliaetus*		- Distance to the nearest nesting site (km)	0	(3)	0	(3)
				2	3	2	3
					Sum of the weighted status grades (left) and of the weighted CI grades (right) of the 13 boxes^a^	107	122
					EBQI^b^ (left) and CI^c^ (right) of the site	6.4	2.9

a The maximum value of the sum is 168 (see text).

b Maximum value: 10 (see text).

c Maximum value: 4 (see text).

CI: Confidence Index. DM: dry mass, including calcium carbonate. HOM: indicators of high level of organic matter. LOM: indicators of low level of organic matter. MPO: Multicellular Photosynthetic Organisms. POM: Particulate Organic Matter. WM: wet mass.

Bad (EBQI<3.5): Sitges.
Poor (3.5≥EBQI<4.5): Niolon, Gulf of Giens and Porquerolles Island northern coast.
Moderate (4.5≥EBQI<6): Villefranche Bay, Saint-Cyr Bay, Plateau des Chèvres (Marseilles), Prado Bay (Marseilles), Valincu Gulf, Tossa de Mar, Scandola (Elbu Bay) and Peyrefite Bay.
Good (6.0≥EBQI<7.5): Espardell and Porquerolles Island southern coast.
High (EBQI≥7.5): Bagaud Pass (Port-Cros Island), Medes Islands and Port-Cros Island (southern coast).

### Redundancy of EBQI with already existing indices

Is the EBQI superfluous, i.e. redundant with already existing indices? A number of indices (EQR, Ecological Quality Ratio) based upon *P. oceanica* (the species itself, sometimes leaf epibiota, not the ecosystem) have been proposed and are currently routinely used, generally for the purpose of monitoring the ecological status of a water body, e.g. POMI [12], PREI [14] and BiPo [15]. They are based on different combinations of a number of parameters, e.g. shoot density, leaf surface area per shoot, depth of the lower limit of the meadow, percent cover of the dead matte and epibiota biomass. One or several of these indices are available from the literature for 13 out of the 17 study sites (Table 6). As these indices are more or less congruent [16], we have investigated the possible correlation between EBQI and the available EQR (POMI, BiPo or PREI) in these 13 sites, and between EBQI and PREI. There is no significant correlation (Spearman coefficient), either when all available EQRs are considered (n = 13, r_s_ = 0.469, p value = 0.11) or when only PREI data are concerned (n = 9, r_s_ = −0.017, p value = 0.97). This result is not unexpected: EQRs mainly assess the health of the seagrass, linked to e.g. the water quality, while EBQI measures the status of the ecosystem, linked not only to water quality but also to e.g. the overfishing. The contrasting ranks of Porquerolles Island (northern coast) through EBQI (poor) and EQR (high, first ranking, see Table 6), together with those of Valincu Gulf and Gulf of Giens, can be due to impacts other than the water quality, e.g. artisanal and recreational overfishing and anchoring of pleasure boats.

**Table 6:** Comparison of EBQI with Ecological Quality Ratios (EQRs) based mainly upon *P. oceanica* (the organism itself) and aimed at establishing the ecological status of the seawater body.

Site	EBQI/10 (CI)	EQR/1	Type of index	Reference
Port-Cros Island, southern coast	9.3 (1.6)	0.802	PREI^a^	[14]
Medes Islands	7.9 (2.4)	0.752	POMI^b^	[12]
Scandola, Elbu Bay	5.7 (2.6)	0.802	BiPo^c^	[15]
Tossa de Mar	5.6 (3.2)	0.682	POMI	[12]
Valincu Gulf	5.4 (1.4)	0.386	PREI	[14]
		0.729	BiPo	[15]
Prado Bay, Marseilles	5.3 (2.3)	0.636	PREI	[14]
Plateau des chèvres, Marseilles	5.0 (3.1)	0.477	PREI	[14]
Saint Cyr Bay	4.9 (0.7)	0.682	PREI	[14]
Villefranche-sur-Mer Bay	4.8 (1.3)	0.280	PREI	[14]
Gulf of Giens	4.3 (2.4)	0.708	PREI	[14]
Porquerolles Island, northern coast	4.3 (2.4)	0.819	PREI	[14]
Niolon (Côte Bleue)	3.9 (1.7)	0.465	PREI	[14]
Sitges	2.3 (2.7)	0.238	POMI	[12]

a The metrics of PREI are: shoot density, shoot leaf surface area, ratio between epibiota biomass and leaf biomass, depth of the lower limit and type of this limit [14].

b The metrics of POMI are: shoot density, meadow cover, percentage of plagiotropic rhizomes, shoot leaf surface area, percentage of foliar necrosis, P, N and sucrose content in rhizomes, δ^15^N and δ^34^S isotopic ratio in rhizomes, N content in epiphytes, Cu, Pb and Zn content in rhizomes [12].

c The metrics of BiPo are: shoot density, shoot leaf surface area, lower depth limit and lower limit type [15].

PREI, POMI and BiPo indices are based upon distinct but similar metrics and range from 0 (lowest ecological status) to 1 (highest ecological status).

### Redundancy of parameters to calculate the EBQI

Are some of the parameters used (functional compartments, boxes) redundant with other boxes or with the EBQI? Should such a redundancy exist, this could mean either that the box alone is sufficient to estimate the whole ecosystem's status, or that some boxes are useless. Obviously, the higher weighting is given to a box, the stronger is the probability of such a correlation. Some boxes (e.g. box 5 ‘Pinna’ and box 10 ‘predators’) are significantly correlated with others and/or with the EBQI (Table 7). However, the removal of the boxes correlated with others (boxes 5, 9, 11 and SRDI) results in changes (loss of accuracy?) in the EBQI: the five-class ranking (see below) of 53% of the study sites moves from one class to the next (result not presented). In addition, the removal of these possibly superfluous boxes would not save field time (data acquisition), since data for these boxes are acquired simultaneously with those of non-removed compartments.

**Table 7:** Spearman's rank correlations between the status of each compartment (boxes 1 through 13 and SRDI), the other compartments (boxes) and the EBQI (see Table 5).

	1.Rhizomes	2. Leaves	3–4. Leaf epibiota	5. Pinna	6. HOM LOM	7. Litter	8. Holothuria	9. Herbivores	10. Predators	11. Piscivores	9–12 (SRDI)	12. Planktivores	13. Sea birds
1. Rhizomes	-												
2. Leaves	0.44	-											
3–4. Leaf epibiota	0.03	0.41	-										
5. Pinna	0.71	0.64	0.02	-									
6. HOM-LOM	0.57	0.33	−0.02	0.63	-								
7. Litter	0.44	0.55	0.29	0.48	0.51	-							
8. Holothuria	0.27	0.27	0.20	−0.09	−0.08	0.44	-						
9. Herbivores	0.07	0.03	0.20	−0.08	0.23	0.67	0.46	-					
10. Predators	−0.02	0.23	0.29	0.37	0.36	0.34	0.02	0.24	-				
11. Piscivores	0.22	0.30	0.33	0.51	0.46	0.42	−0.22	0.10	0.73	-			
12. Planktivores	0.18	0.28	0.38	0.34	0.24	0.34	0.05	0.06	0.48	0.62	-		
9–12 (SRDI)	0.16	0.32	0.30	0.56	0.59	0.41	−0.02	0.29	0.85	0.60	0.46	-	
13. Sea birds	0.37	0.22	0.37	0.12	0.19	0.36	0.30	0.26	0.20	0.18	0.08	0.19	-
EBQI	0.45	0.69	0.50	0.72	0.55	0.78	0.24	0.40	0.69	0.65	0.54	0.77	0.47

Bold italic characters: significant (p<0.01).

### Robustness of the EBQI

**Figure 2:**
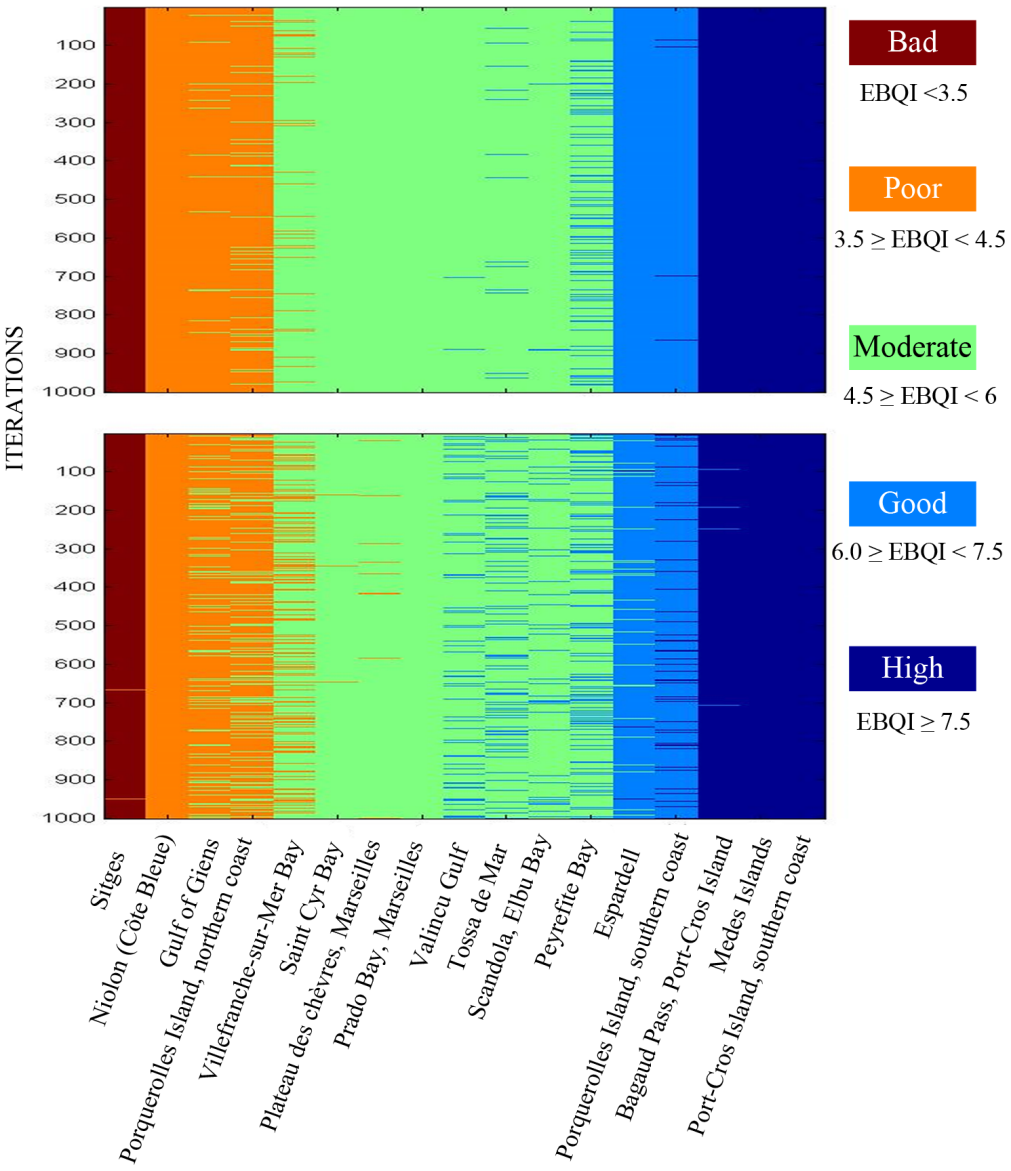
Robustness of the quality index (EBQI) with regard to the weighting of compartments (boxes). The 17 sites are arranged from left to right according to their growing EBQI (see Table 4) and ecological status (bad through high). Deep red = bad, orange = poor, green = moderate, light blue = good, deep blue = high. In order to test the effect of the weighting of the compartments (boxes) on the EBQI (robustness), weighting values have been randomly perturbed (above, ±1; below, ±2). 1000 iterations were performed. The change of the ecological status (bad through high) of a site, for a given iteration, is shown by the color of the new class within which it falls.

**Figure 3:**
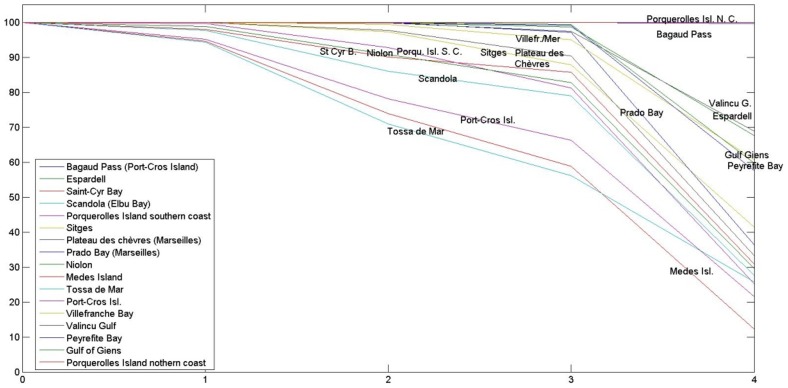
Robustness (index of similarity) of the quality index (EBQI) to the weighting of compartments (boxes). Percentage of times the ecological status of each site was unaltered by random perturbation of the weighting of the boxes (1,000 iterations) by ±1 through ±4. The similarity is equal to 100% when the class of the site is never changed after perturbation and 0% if it is always changed.

Is the EBQI robust, i.e. only slightly influenced by arbitrary choices in its conception and biases? Possible arbitrary choices are e.g. choice of the considered compartments (boxes) and the weighting (1 through 5) of the boxes. As far as the choice of the compartments taken into account is considered, it should be first emphasized that the choice is anything but arbitrary; boxes encompass the whole spectrum of an ecosystem functioning, from primary producers, herbivores, filter-feeders, suspension-feeders and detritus-feeders to top predators (Fig. 1). It is worth noting that the removal of up to 4 boxes (see above) only slightly changes the five-class ranking of the study sites: at the most, half of the sites move from one class to the next (e.g. good to moderate, poor to bad; results not presented). Finally, the changing of the weighting of the boxes (hereafter ‘perturbation’) alters the ranking of the sites; the changes increase with the importance of the perturbation (±1, ±2, fig. 2; ±3 and ±4, not presented). However, the changes due to weighting perturbation are relatively slight. In addition, an index of similarity was produced for each site, which is equal to 100% when the rank of the site is always unchanged after perturbation and 0% if it is always changed (Fig. 3). Some sites are very robust (e.g. Porquerolles Island northern coast and Bagaud Pass), other are more sensitive to the choice of weightings (e.g. Medes Islands and Tossa de Mar). The similarity ranges between 100 and 95% (perturbation ±1), 100 and 71% (±2), 100 and 57% (±3) and 100 and 12% (±4). The low values of the similarity index for high levels of perturbation of the weighting emphasize the fact that weighing the boxes is useful, despite the robustness of this parameter.

### EBQI implementation

Is the EBQI excessively time-consuming or easy and rapid to implement? The acquisition of the parameters requires SCUBA diving field work. Considering that it is possible to work for around 1 hour at 15 m depth, we estimate that 6 dives involving 2 scientific divers are necessary to acquire all of the data for the assessment of one site (Table 8). For safety reasons, a diver can perform a maximum of two dives per day, so this will require 3 days of field work for a two man diving team. This field work requires expertise in seagrass and fish visual censuses. In addition, for a rapid and provisional assessment of the EBQI, already available data and expert judgment can be used (provided that the CI is specified).

**Table 8:** Estimation of time and diving effort needed for data acquisition within each box used in the EBQI.

Box	Proxy	Time	Dive organization
1 – *P. oceanica* roots and rhizomes	30 random *in situ* measures (growth of the rhizome corresponding to the last 8 leaf bases)	30 min	1 dive for 2 scientific divers
2 - *P. oceanica* leaves	20 random measures of shoot density in a square frame (0.16 m^2^)	60 min	
	30 random measures of cover	10 min	
3 - MPO leaf epibiota	Sampling of the two oldest external leaves on 30 shoot randomly localized to estimate the epibiota biomass	10 min	
4 - Filter- and suspension-feeder leaf epibiota	Sampling of the two oldest external leaves on 30 shoot randomly localized to estimate the epibiota biomass	Same as box 3	
5 – Filter feeder benthic epibiota	Density of *Pinna nobilis* along 20 transects 10-m long and 1-m wide	120 min	1 dive for 2 scientists divers
7 - Litter detritus	Litter detritus mass collected in July, within 5 randomly localized 0.1-m^2^ quadrats	90 min	1 dive for 2 scientist divers
6 – Other filter- and suspension-feeder benthic epibiota	Abundance of filter- and suspension-feeder benthic epibiota other than *Pinna nobilis* within 1-m^2^ quadrats, with 30 replicates randomly localized	120 min	1 dive for 2 scientist divers
8 - Detritus feeders	Abundance of *Holothuria* spp. within 1-m^2^ quadrats, with 30 replicates randomly localized	Same as box 6	
9 - Herbivores 1	Abundance of *Paracentrotus lividus* within 1-m^2^ quadrats, with 30 replicates randomly localized at 5 m depth	60 min	1 dive for 2 scientist divers
	Percentage of intermediate and adult leaves exhibiting bite scars due to *S. salpa* (all the intermediate and adult leaves of 30 shoots randomly localized) at 5 m depth	30 min	
10 - Predatory teleosts	All teleosts counted within ten linear 50-m long and 5-m wide transects, each census lasting 5 minutes.	60 min	1 dive for 2 scientist divers
11 - Piscivorous teleosts	All teleosts counted within ten linear 50-m long and 5-m wide transects, each census lasting 5 minutes	Same as boxes 10 and 12	
12 - Planktivorous teleosts of the water column	All teleosts counted within ten linear 50-m long and 5-m wide transects, each census lasting 5 minutes	Same as boxes 10 and 11	
9–12 - SRDI	All teleosts counted within ten linear 50-m long and 5-m wide transects, each census lasting 5 minutes	Same as boxes 10, 11 and 12	

Within the northern Mediterranean, the study sites are spread within three eco-regions, namely Corsica, Provence and French Riviera and Languedoc-Catalonia. Is the EBQI biased by regional environmental conditions, e.g. a higher mean temperature in Corsica and more water turbidity in Languedoc-Catalonia? The present study cannot answer this question as the sites were not randomly selected but chosen according to data availability. However, the EBQI range within each one of the three eco-regions does not exhibit obvious differences (Table 4), as far as the EBQI mean is concerned (∼5.6 within each one of the three eco-regions).

## Discussion

In the context of European Directives (Habitats Directive and WFD) and national regulations, a number of species and indices, based upon one or several species, have been designated to assess the quality of water bodies, the health status of emblematic species and habitats and to establish Marine Protected Areas (e.g. PMN [112], CI [113], EEI [2], [3], SI [114], POMI [12], PREI [14], BiPo [15], PoSte [15], ZoNI [22] for European seagrasses ([17], [51], [115], [116] for comprehensive reviews). These indices provide a valuable body of tools and information to coastal waters managers and make it possible to assess the status of a water body. However, the good quality of a water body and the apparent health of a species, whether emblematic or not, such as the seagrass *P. oceanica*, is not always indicative of the good structure and functioning of the whole ecosystem, a network of compartments, fluxes, functions, inputs and exportations. The most original contribution of the new MSFD European Directive is to provide an ecosystem-based approach to assess the ecological status of marine regions. The present approach constitutes a contribution towards this goal, and focuses on the *P. oceanica* seagrass meadow.

Here, we have developed, applied and tested an ecosystem-based index of the structure and functioning of the most emblematic and best-known Mediterranean coastal ecosystem, the *P. oceanica* meadow. This index (EBQI) is based upon a set of representative functional compartments, the weighting of these compartments and the assessment of each compartment quality by comparison of a supposed baseline based upon the available literature. The strong points of the EBQI are: **(i)** It is easy to implement, not too time-consuming and therefore relatively cheap; **(ii)** It is non-destructive, which is particularly important, dealing with protected species (e.g. *P. oceanica* and *Pinna nobilis*); sampling only concerns old leaves (not the shoot) and the litter, i.e. shed dead leaves; **(iii)** It is relatively robust, according to the selection of the compartments and to their weighting; **(iv)** It is associated with confidence indices CI (each compartment, and the overall mark) which indicate possible weakness and biases and therefore the need for further field data acquisition; **(v)** It can draw the attention of managers to sites whose CI is high and where routine monitoring can therefore be implemented, and conversely those with a low CI indicating a lack of knowledge.

The Integrated Coastal Zone Management (ICZM) contributes towards maximizing the benefits provided by the coastal zone and minimizing conflicts and the harmful effects of activities upon each other (e.g. [117]). ICZM, together with Ecosystem-Based Management (EBM; e.g. [118]) and, more generally, the implementation of European Directives, are in need of indices to assess ecological quality, either based upon a single or a few species, or ecosystem-based. The sites corresponding to well implemented MPAs, such as the Port-Cros National Park (Port-Cros Island southern coast, Bagaud Pass) and the Medes Islands marine reserve, not unexpectedly get the best marks, which is congruent with the overall excellent status of their habitats [108]. In contrast, sites localized in areas highly impacted by most human activities and waste, such as Sitges (South of Barcelona) and Niolon (close to the port of Marseilles and outfall of wastewater), obtain a very low mark. Though sites with obviously good or bad ecosystem status naturally obtain a good and bad, EBQI mark, respectively, some sites (e.g. Saint-Cyr Bay and Villefranche-sur-Mer Bay) do not obtain the mark they would seemingly have deserved through the apparent health of the seagrass itself. This confirms the usefulness of an index, EBQI, based upon the whole ecosystem rather than only upon the seagrass (sometimes also epibiota and other parameters). It is worth noting that the Confidence Index (CI) is overall relatively low. This is especially the case of Saint-Cyr Bay (CI = 0.7; table 4) and Villefranche-sur-Mer Bay (CI = 1.3), which could also account for the surprisingly low and not so low, EBQI, respectively, of these sites, according to literature data [119], [120].

The weak points of the EBQI are: **(i)** The baselines used to assess the compartments, which may prove to be biased either by the poor knowledge or availability of totally non-human impacted areas, or by climate change which renders the supposed baseline obsolete; **(ii)** The probable need for testing and/or adapting the EBQI in areas distant from the study area -the north-western Mediterranean- and more generally to other seagrass ecosystems; (iii) The conceptual model which constitutes the basis for the present ecosystem-based approach is obviously oversimplified. For example, small-sized predatory ‘invertebrates’ were not taken into consideration. Some of them belong to the matte endofauna box, which encompasses detritus feeders, suspension-feeders and predators. Other are creeping or clinging organisms on the *P. oceanica* leaves. However, considering them would have made the model more complex and weighed down the assessment of ecosystem status. Moreover, other compartments are likely to provide redundant information, so that no improvement in accuracy is to be expected.

A number of attempts to review and compare the biotic indices using benthic (e.g. macrophytes) and pelagic marine, lagoon and estuarine organisms, have been performed [16], [39], [51], [116], [121]. For example, [116] analysed the strengths and weaknesses of 90 published indices. They identified several weaknesses: **(i)** problems of applicability due to practical and conceptual difficulties, affecting most of the indices related to ecosystem function, **(ii)** the failure of many indices using e.g. the taxonomic composition of the community to connect its condition with the stressors, and **(iii)**, as far as indices based upon the sub-individual level are concerned (e.g. multi-biomarkers), their poor strength to assess the ecological integrity of the habitat. They concluded that the most promising approach would be to aggregate indices with complementary strengths. In a sense, the EBQI could be considered as such an aggregative index. Although the goal of the EBQI is not to compete with the already available biotic indices utilizing the *P. oceanica* seagrass meadow for assessing the quality of a water body, but to assess the status of the proper seagrass ecosystem, the comparison of the EBQI with some other biotic indices will be of relevance. An attempt has been made here, using some published data; a more accurate comparison would require data acquisition at the same sites as those used for the EBQI, and is therefore beyond the scope of the present work. In addition, the correlation between the EBQI and anthropogenic gradients, including overfishing, will be useful, in order to assess which human impacts are of major relevance for the status of the ecosystem. This will constitute the next step.

## Conclusions

Overall, the Ecosystem Based Quality Index (EBQI) is easy to implement, relatively robust and does not seem to be redundant with existing indices based upon the seagrass itself. In addition, the non-congruence of the EBQI, i.e. the quality of the ecosystem functioning, with the empirical idea one may have of the *P. oceanica* ecosystem at a given site, due e.g. to the health of the seagrass, the clearness of the water (and even the beauty of the landscape and the seascape) confirms the usefulness of the EBQI index, based upon the whole ecosystem rather than upon only the seagrass. Other anthropogenic impacts, e.g. overfishing, are putatively more important than the above mentioned parameters for the structure and functioning of the ecosystem. Finally, the EBQI that is proposed here for the *P. oceanica* ecosystem could constitute a model for similar indices designed for other ecosystems, such as coralligenous outcrops, underwater caves, soft bottoms and sublittoral reefs.
